# Taenia solium in the Anterior Chamber: Surgical Management of a Rare Case of Live Free-Floating Parasite

**DOI:** 10.1155/2021/4038691

**Published:** 2021-09-30

**Authors:** Amrit Banstola, Sweta Singh, Sarita Maharjan, Gyanendra Lamichhane, Anadi Khatri

**Affiliations:** ^1^Lumbini Eye Institute and Research Center, Lumbini, Nepal; ^2^Birat Eye Hospital, Biratnagar, Nepal; ^3^Department of Ophthalmology, Birat Medical College and Teaching Hospital, Biratnagar, Nepal

## Abstract

Ocular cysticercosis is a preventable cause of blindness. It is a parasitic infestation caused by Cysticercus cellulosae—which is the larval form of Taenia solium. In 1829, Soemmering reported the first case of a live anterior chamber cysticercosis. In the following, we report a rare case of a 13-year-old male who presented with a live adult Taenia solium in the anterior chamber without any systemic features and its successful management.

## 1. Introduction

Ocular parasitosis continues to intrigue ophthalmologists with a variety of presentations [1]. They can remain obscure and continue to manifest as a “masquerade” until specifically searched for. Even when identified, it provides a challenge to the treating physician to decide on how to proceed further [2–4]. One of such similar parasitic infestations is cysticercosis.

Cysticercosis parasitic infection/infestation of human tissue is caused by the cystic form of the larvae of Taenia solium [5]. It gains entry into the human body via a faeco-oral route as a result of consumption of contaminated food/water [6]. While adult worms are mainly responsible for inducing malnutrition and intestinal obstructions, the larvae (cysticerci) can produce more life-threatening conditions. It has the potential to invade muscles, subcutaneous tissues, and central nervous system and is in fact one of the major causes of adult-onset seizures in low-income countries [1, 5, 7, 8]. Eyes and adnexal structures such as eyelids, orbit, extraocular muscles, subconjunctival space, anterior chamber, vitreous, and subretinal space are affected in 13-46% of infected individuals [8]. Ocular manifestations can be devastating if the cysticercoses increase in size or die releasing toxic products leading to profound inflammatory reaction causing blindness in 3-5 years [9]. We here report an extremely rare case of a live adult Taenia solium in the anterior chamber and also discuss on how it was successfully managed.

## 2. Case Report

A 13-year-old boy presented with complaints of progressive diminution of vision associated with redness in the left eye for 9 days. The episode was not associated with pain and discharge. The patient denied seeing floaters or shadows. Otherwise healthy, the patient also denies history of trauma, similar history in the past, or use of any topical medication. He was a student by profession but occasionally helped in feeding the livestock of the family—which involved cows and buffalos. The patient gives no history of consumption of undercooked meat. The patient and his party also denied any history of abnormal body movement or seizure till date. The rest of the history was found to be insignificant. Visual acuity and intraocular pressure of the affected eye were hand movements close to face and 21 mmHg, respectively. The findings of the unaffected eye were found to be normal.

**Figure 1:**
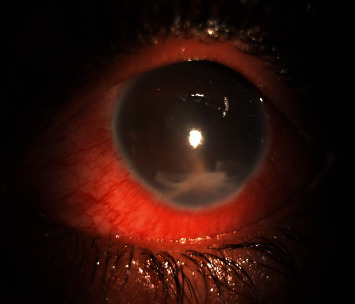
Clinical picture of the affected eye. Note the hazy cornea, circumciliary congestion, and the worm along with exudates in the inferior angle.

On slit-lamp examination, the conjunctiva was found to be congested with circumciliary congestion and hazy cornea (Figure 1). On evaluation of the anterior chamber, exudative infiltrates with the presence of a mobile and slender tubular worm-like intraocular foreign body were visible at 6 o'clock. The entity responded to increased intensity of the light from the slit lamp with increased mobility—episodic elongation and curling. Right eye examination was within normal limits.

Detail fundus examination could not be done due to hazy cornea and the presence of dense exudates in the anterior segment chamber. Ultrasonography (USG) of the left eye was done, and the posterior segment was found to be radiologically normal.

Routine blood counts with differential/absolute eosinophil counts were unremarkable. Stool examinations revealed no ova, cysts, or adult worm. USG of abdomen and pelvis were unremarkable. The patient was also advised for non-contrast-enhanced computerized tomography (NECT) of the head and orbit which yielded normal reports. The patient was planned for surgery to remove the worm via viscoexpulsion and parasitological examination.

### 2.1. Surgical Procedure and Postoperative Management

**Figure 2:**
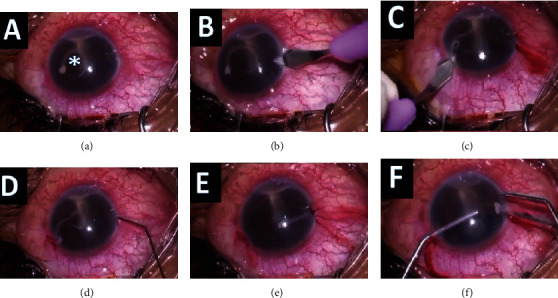
(a) Worm in the anterior chamber (marked with asterisk ∗). (b, c) Clear corneal incisions made at 9 and 2 o'clock. (d) Injecting pilocarpine 0.5% intracamerally. (e) Paralyzed worm which has coiled up. (f) Worm removed in toto with the use of a viscoelastic device from 2 o'clock port and expression from 9 o'clock port.

The patient was prepared for surgery under peribulbar anesthesia. After painting and draping, two 2.8 mm clear corneal incisions were made at 9 o'clock and at 2 o'clock positions (Figures 2(a)–2(c)). Pilocarpine nitrate 0.5% was injected into the anterior chamber (Figure 2(d)). This helps in constricting the pupil and is also a chemoparalyzing agent which can help in decreasing the movement of the worm [3, 10].

Once the worm was observed to be hypomobile, the anterior chamber was injected with viscoelastic surgical device (VSD) from the corneal port created at 2 o'clock to push the worm and the exudates away from the pupil (Figure 2(e)). The port at 9 o'clock was opened using angled plain forceps (Figure 2(f)). The worm was viscoexpulsed in toto using the no-touch technique. The worm was then transferred into a bottle containing normal saline and transferred to the microbiology and parasitology department for identification.

### 2.2. Postoperative Management and Follow-Up

**Figure 3:**
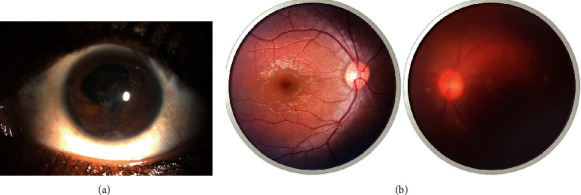
(a) Anterior segment photograph at 4^th^ postoperative week. A large patch of iris pigment can be observed over the pupillary axis over the anterior capsule. Posterior synechia remained from 2 to 7 clock hours. Opacification of the anterior capsule was also present on the central region. Also, note an anterior synechia at 6 o'clock—where the worm was residing presurgically. (b) Fundus photo of the right eye (normal) and the left eye (affected) at 4^th^ week of follow-up. No obvious vitreous or chorioretinal lesions were seen. Note the hazy fundus picture due to media opacity over the anterior lens capsule.

The patient was started on oral prednisolone 1 mg/kg/day and also given a single dose of 400 mg albendazole. On the first postoperative day, the VA of the patient was still hand movement with intraocular pressure of 14 mmHg. The anterior chamber still had signs of inflammation while the posterior segment looked normal echogenically. The patient was started on topical moxifloxacin 0.5% and prednisolone acetate 1%—both 2 hourly—and tropicamide 1% thrice daily. He was advised to follow up in a week. At the first follow-up, the patient had best-corrected visual acuity of 6/36 and the anterior chamber inflammation had subsided. The patient was advised to continue the same medications with steroids being tapered on weekly basis. The patient was advised to follow up after 4 weeks. At the second follow-up, the patient presented with the best visual acuity of 6/18. Anterior segment examination revealed iris pigmentary patches over the pupillary axis secondary to medical synecholysis and anterior synechiae at 6 o'clock but without inflammation (Figure 3(a)). Due to media opacity, the posterior segment findings looked hazy but retinal and optic disc structures could be appreciated and appeared normal (Figure 3(b)). All his medications were stopped, and he was kept under regular follow-ups for observation.

### 2.3. Microbiological and Parasitological Findings

**Figure 4:**
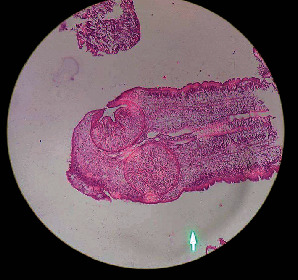
Microbiological assessment of the parasite. Note the refractile cuticle with reticular tissue. The subcuticular layer consisted of aggregated subcuticular cells, small myofiber bundles with scolex, two suckers, and birefringent four hooklets.

On histopathological examination using hematoxylin and eosin stain, a translucent flexible worm measuring 11 × 1.5 mm was observed. There was presence of an outer refractile cuticle with reticular tissue. The subcuticular layer consisted of aggregated subcuticular cells, small myofiber bundles with scolex, two suckers, and birefringent four hooklets consistent with Taenia solium (Figure 4).

## 3. Discussion

Taeniasis, the condition caused by infection with adult worm, Taenia solium, is worldwide in distribution, but endemic in some parts of the world [7]. While taeniasis is rarely seen in those who do not eat pork, cysticercosis occurs in all ethnic groups regardless of dietary habits. In the normal life cycle of T. solium, man is the definitive host and pig is the intermediate host. Cysticercosis is known to involve ocular tissues—mainly intraocular structures (anterior and posterior segment) and extraocular tissues (extraocular muscles, orbit, subconjunctival space, eyelids, optic nerve, and lacrimal gland) [1].  Table 1 enlists the findings from the relevant existing literature regarding its manifestations in the eye when present in the anterior chamber.

Anterior chamber cysticercosis is an unusual presentation, and the occurrence of a live mobile parasite in the anterior chamber is a rarer occurrence. The route entry of the cyst in the anterior chamber is inconclusive so far, but it has been suspected to enter the anterior chamber from the posterior segment through the pupil in aphakes, through vessels supplying the ciliary body, or through the anterior chamber angle. [17].

As with any other parasitic infestation in the ocular tissues, the inflammation is usually very intense. There may be pain and redness with associated iridocyclitis or glaucoma [16]. Glaucoma may be inflammatory in the presence of iridocyclitis or due to pupillary block caused by the cyst. Surgical management by removal of the parasite is the treatment of choice for all live cases. The most commonly adopted method is the “no-touch technique with viscoexpression” [2, 3, 18]. For a mobile worm, there is often a risk of migration to the posterior chamber via the pupil during the surgical procedure. Various chemoparalytic agents like pilocarpine and carbachols that also have miotic properties can be of great advantage in such cases [3, 10].

However, it must be noted that parasites, if dead, may not elicit any inflammation [4]. Such cases have been reported to be well managed simply by observation. It may be advisable to approach such cases as per surgeons' preference. In case of a decision for surgical removal, care must be taken not to disrupt the capsule or the outer coating of these worms which may lead to anaphylaxis-like reaction.

It is of utmost importance that we enlist the possibility of intraocular parasites and specially cysticercosis as a case presenting with atypical uveitis. The clinical suspicion and diagnosis of live intraocular cysticercosis are based on the morphology of the parasite and hence removal in toto followed by proper storage, transportation, and microbiological examination which remains critical for correct diagnosis [6].

For every intraocular parasitosis, systemic treatment with antihelminthic and steroids is often always recommended. But more importantly, systemic manifestations must always be ruled out. Stool examination, high-resolution ultrasonography (USG), computed tomography (CT), and magnetic resonance imaging (MRI) can help in the identification and management of such cases [19].

## 4. Conclusion

Surgical removal of the live parasite is the mainstay of treatment. Medical therapy is recommended for a duration of at least 1 month with albendazole and steroids given the complexity of route of migration for the organism to reach the eye. A high level of suspicion for probable involvement of other systemic tissues, such as the central nervous system, must always be considered, and systemic treatment is considered to alleviate the benefit of doubt.

## Figures and Tables

**Table 1:** Review of relevant published articles on anterior segment cysticercosis along with their common findings, management, and outcome.

Authors	Findings	Management	Outcome
Soemmerring et al. (1829) [11]	—	Surgical removal	—
Mathur (1962) [12]	Cystic form in anterior chamber, with scolex buried in the iris. Plastic iridocyclitis with hypopyon.	The cyst was removed from the anterior chamber by an ab extemo incision of the cornea from 2-5 o'clock.	Iridocyclitis cleared up early, and the patient's vision improved to 6/12 from counting of fingers at 1 meter.
Welsh and Proctor (1978) [13]	Anterior and intermediate uveitis with inferior exudate in the anterior chamber. Worm was observed within the exudate.	Removal via corneal incision and placement of curette. Oral and topical steroids.	Hypotonic eye with vascularization of the iris and the lens.
Das et al. (2002) [14]	Extension of taenia cellulosae into both ant and post segment of the eye.	Surgical management using viscoexpression and suturing of the corneal incision. Coverage with systemic steroids and albendazole.	6/12 at the time of presentation. 6/18 at the time of follow-up after the surgery.
Chandra et al. (2007) [15]	Cyst in the anterior chamber with occasional protrusion and retraction of the head/scolex by the organism.	Viscoexpression followed by topical steroid coverage.	6/36 at the time of presentation. No relatable complications at follow-ups after the surgery.
Mahendradas et al. (2007) [16]	Multiple cysts of T. cellulosae in the anterior chamber. Plastic uveitis with ocular hypertension.	Viscoexpression followed by treatment with topical antibiotics, steroids, and antiocular hypertensive agents.	6/12 at the time of presentation. 6/6 after treatment.
